# Telomere Length and Genetic Anticipation in Lynch Syndrome

**DOI:** 10.1371/journal.pone.0061286

**Published:** 2013-04-23

**Authors:** Nuria Seguí, Marta Pineda, Elisabet Guinó, Ester Borràs, Matilde Navarro, Fernando Bellido, Victor Moreno, Conxi Lázaro, Ignacio Blanco, Gabriel Capellá, Laura Valle

**Affiliations:** 1 Hereditary Cancer Program, Catalan Institute of Oncology, IDIBELL, Hospitalet de Llobregat, Barcelona, Spain; 2 Unit of Biomarkers and Susceptibility, Catalan Institute of Oncology, IDIBELL and CIBERESP, Hospitalet de Llobregat, Barcelona, Spain; 3 Department of Clinical Sciences, Faculty of Medicine, University of Barcelona, Hospitalet de Llobregat, Barcelona, Spain; IFOM, Fondazione Istituto FIRC di Oncologia Molecolare, Italy

## Abstract

Telomere length variation has been associated with increased risk of several types of tumors, and telomere shortening, with genetic anticipation in a number of genetic diseases including hereditary cancer syndromes. No conclusive studies have been performed for Lynch syndrome, a hereditary colorectal cancer syndrome caused by germline mutations in the DNA mismatch repair genes. Here we evaluate telomere length in Lynch syndrome, both as a cancer risk factor and as a mechanism associated with anticipation in the age of cancer onset observed in successive generations of Lynch syndrome families. Leukocyte telomere length was measured in 244 mismatch repair gene mutation carriers from 96 Lynch syndrome families and in 234 controls using a monochrome multiplex quantitative PCR method. Cancer-affected mutation carriers showed significantly shorter telomeres than cancer-free mutation carriers. In addition, cancer-affected carriers showed the most pronounced shortening of telomere length with age, compared with unaffected carriers. The anticipation in the age of cancer onset observed in successive generations was not associated with telomere shortening, although, interestingly, all mother-son pairs showed telomere shortening. In conclusion, cancer-affected mismatch repair gene mutation carriers have distinct telomere-length pattern and dynamics. However, anticipation in the age of onset is not explained by telomere shortening. Pending further study, our findings suggest that telomere attrition might explain the previously reported dependence of cancer risk on the parent-of-origin of mismatch repair gene mutations.

## Introduction

Lynch syndrome (LS) (MIM 120435) is the most common hereditary syndrome that predisposes to colorectal cancer (CRC) and other extracolonic tumors, accounting for 2%–5% of the total burden of CRC. It is caused by germline mutations in the DNA mismatch repair (*MMR*) genes *MLH1* (MIM 120436), *MSH2* (MIM 609309), *MSH6* (MIM 600678) and *PMS2* (MIM 600259), as well as in *EPCAM* (MIM 185535). There is large phenotypic variation in the age of onset among and within families [1], [2]. Progressively earlier age of cancer onset in successive generations has been reported [3]–[10]. However, the underlying molecular mechanisms of both the overall phenotypic variation and the anticipation in the age of onset are still unknown.

Telomere shortening has gained considerable interest as a potential biomarker of cancer risk and as a mechanism associated with genetic anticipation. Moreover, we have recently shown that a common genetic variant located in the telomerase gene (*hTERT*; MIM 187270) increases cancer risk in LS patients at early ages, and that this SNP is associated with shortened telomere length in cancer-affected *MMR* gene mutation carriers [11]. Telomeres consist of multiple short repeats (TTTAGG) at the ends of chromosomes and protect them against large-scale genomic rearrangements. In most somatic cells, telomeres shorten with each replication cycle, eventually leading to cellular senescence or apoptosis. Interestingly, telomere length anomaly appears to be one of the earliest and most prevalent genetic alterations in the process of malignant transformation [12], [13]. Given the proposed role of telomere shortening in early processes of carcinogenesis, attention has been directed to its potential role as a cancer risk biomarker. With this purpose, a number of retrospective and prospective observational studies have been conducted, although results are inconsistent among studies and tumor types [14]–[16]. With respect to CRC, despite the conflicting results obtained in retrospective studies, prospective studied have not found a convincing association between leukocyte telomere length and CRC risk [16]–[18].

Telomere length attrition has also been proposed as a mechanism of anticipation in different inherited diseases, being associated with early onset and/or severity of disease in genetic disorders such as dyskeratosis congenita [19]–[21], Li-Fraumeni [22], [23], and familial breast and ovarian cancer [24], [25].

Here we aim to elucidate the role of telomere length in LS-associated cancer risk and to study its implication in anticipation in the age of onset observed in successive generations of LS families.

## Materials and Methods

### Ethics statement

Written informed consent was obtained from all subjects. The study received the approval of the IDIBELL Ethics Committee (ref. PR221/09).

### Study participants

A total of 388 individuals, 244 *MMR* gene mutation carriers and 144 non-carriers, from 96 LS families were included in the study. They were assessed through the Hereditary Cancer Program at the Catalan Institute of Oncology, ICO-IDIBELL, from 1998 to 2012. *MMR* gene mutation analysis was performed on genomic DNA extracted from peripheral blood. Large genomic alterations in the genes were studied using Multiplex Ligation-dependent Probe Amplification (MLPA) (SALSA MLPA Kits, MRC-Holland, The Netherlands). Mutation screening was carried out by direct sequencing after PCR amplification (primers and conditions available upon request). Ninety cancer-free individuals were included as controls. All individuals are of Caucasian origin. Informed consent was obtained from all subjects, although data were analyzed anonymously. Table 1 shows a summary of the genetic and clinical characteristics of the groups studied.

**Table 1:** Genetic and clinical characteristics of the studied groups.

	Lynch syndrome families (n = 96)			
			Controls (n = 234)	
	*MMR gene mutation carriers (244)*		*Non-carriers*	*Unrelated controls*
	*Cancer	Cancer-free		
N	144	100	144	90
*MMR* gene mutated: n (%)	*MLH1*: 87 (60.4)	*MLH1*: 62 (62.0)	-	-
	*MSH2*: 42 (29.2)	*MSH2*: 29 (29.0)	-	-
	*MSH6*: 9 (6.3)	*MSH6*: 9 (9.0)	-	-
	*PMS2*: 5 (3.5)	*PMS2*: 0	-	-
	*EpCAM*: 1 (0.7)	*EpCAM*: 0	-	-
Median age at blood draw (± SD)	51.5 (±13.4)	35.0 (±11.5)	42.0 (±14.9)	41.5 (±15.1)
Sex: n (%)	M: 74 (51.4)	M: 40 (40.0)	M: 64 (44.4)	M: 28 (31.1)
	F: 70 (48.6)	F: 60 (60.0)	F: 80 (55.6)	F: 62 (68.9)
Median age at cancer diagnosis (± SD)	43.0 (±12.9)			

N: number of subjects; SD: standard deviation; M: male; F: female.

* Cancer: Individual affected with a LS-associated cancer: CRC and/or cancer of the endometrium, ovary, stomach, small bowel, hepatobiliary tract, pancreas, upper uro-epithelial tract or brain.

Cases and controls were recruited from the same homogeneous population, and storage and DNA extraction from peripheral blood were performed at the same facility and using the same extraction methods. We have observed that blood DNA samples from different sources (i.e., extracted at different laboratories, using different extraction methods, etc.) show large non-genetic variation in telomere length (data not shown). Thus, only samples (cases and controls) that underwent the exact same DNA extraction protocol (Flexigene DNA kit, Qiagen, Hilden, Germany) performed by the personnel of the Unit of Molecular Diagnostics of the Hereditary Cancer Program at ICO-IDIBELL, and that were stored under the same conditions, were included in the study, as recommended by Prescott et al. for retrospective studies [16].

### Telomere length assessment

Telomere length quantification was performed using the monochrome multiplex quantitative PCR method described by Cawthon et al. [26], which has been found to provide greater consistency than other methods used to measure telomere length [17]. The assays were performed using the Quantifast SybrGreen PCR Master Mix (Qiagen, Hamburg, Germany), beta-globin as single copy gene, and the LightCycler 480 real-time PCR detection system (Roche Diagnostics GmbH, Mannheim, Germany). All samples were assayed in triplicate. A standard curve with 7 concentrations spanning an 81-fold rage (60 ng, 40 ng, 20 ng, 6.7 ng, 4 ng, 2.2 ng and 0.74 ng), also in triplicate, of an anonymous standard DNA (healthy and cancer-free 45 year-old individual) was included in every 384-well plate. Good replicates of the standard curves (for telomeres (T) and single copy gene (S)) and subsequent fitted linear regression lines were obtained among plates. Whenever possible, equal numbers of samples from different clinical groups were run in each 384-well plate. The relative telomere length (RTL) value for each sample was calculated according to the standard lines (for T and S) of the corresponding plate. Therefore, the value obtained was relative to the value of the anonymous standard DNA in the same plate (inter-run calibration). The average telomere length of each sample is expected to be proportional to its RTL.

### Statistical analyses

Shortening of telomere length is observed with increasing age in cancer-free individuals [27]. In our control set (controls plus non-carriers, n = 234), RTL was inversely correlated with age (Pearson's correlation r = −0.187; p = 0.004). As previously described, RTL measurements were adjusted for age using the line of best fit for controls [25]. Thus, the difference between the observed and the predicted value was calculated for each sample and is represented as “age-adjusted RTL”. There were no telomere length differences between sexes (data not shown); therefore, sex was not included as a confounding factor in the statistical tests.

Differences in age-adjusted telomere lengths were analyzed using the Wilcoxon rank sum test (Mann-Whitney U). Pearson's test was used to assess the correlation between telomere length and age (telomere length dynamics). To measure the significance of the difference between two correlation coefficients (Pearson's r), Fisher's r-to-z transformation was performed. Anticipation was represented by Kaplan-Meier curves and the differences between survival curves were studied using a log-rank test. Proportions were compared by Fisher's exact test (expected cell count <5) or Chi-square. All tests were two-sided and p-values below 0.05 were considered statistically significant. The analyses were performed using R.

## Results

### Telomere length and shortening dynamics

**Figure 1:**
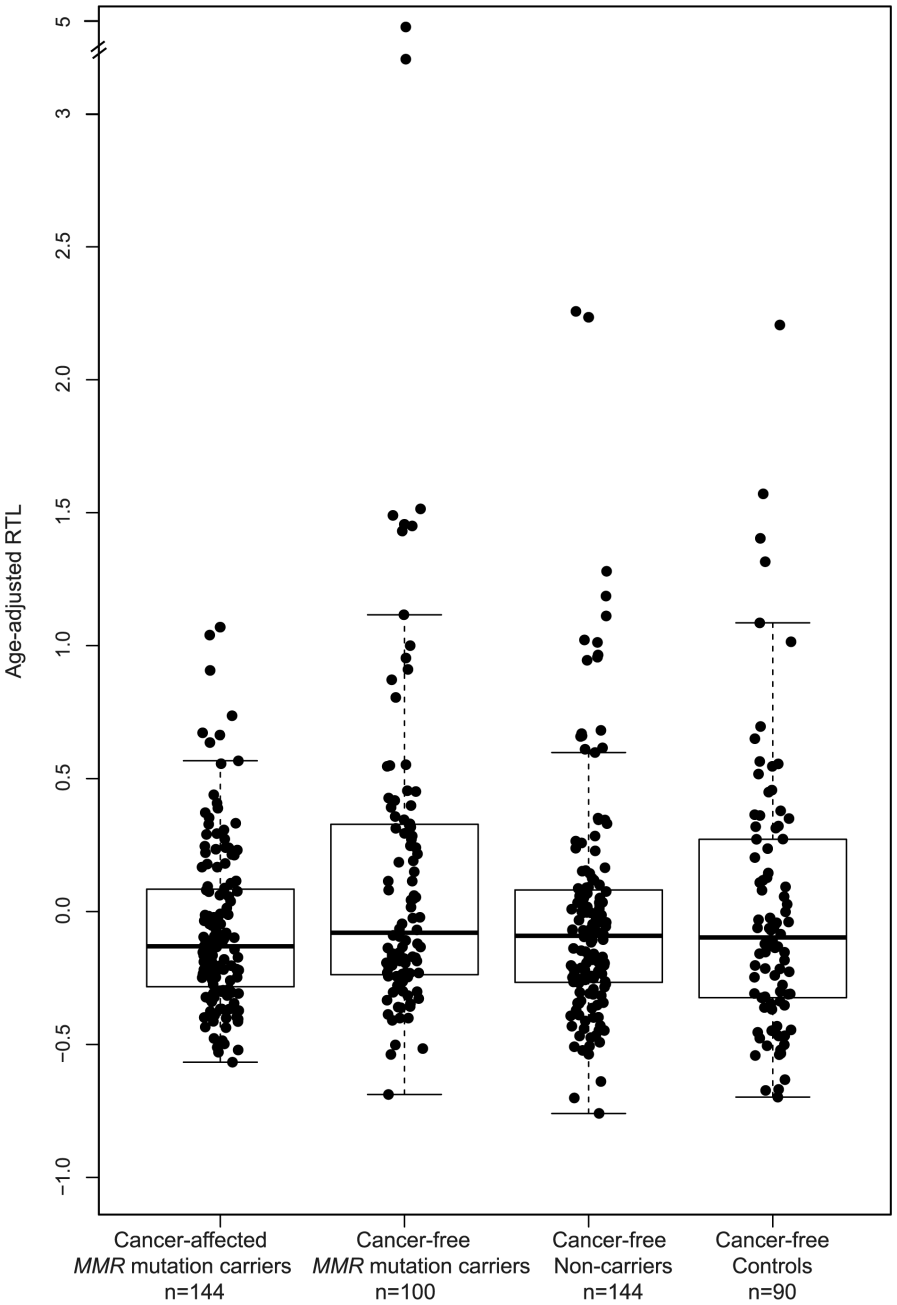
Age-adjusted relative telomere length. In subjects belonging to LS families: *MMR* gene mutation carriers affected with cancer (median: −0.131), *MMR* gene mutation carriers with no diagnosed cancer (median: −0.079) and cancer-free non-carriers (median: −0.091); and in cancer-free controls (median: −0.097). The boxes represent the interquartile range of distributions (25^th^ and 75^th^ percentiles); the horizontal lines within the boxes, the medians; and the vertical lines, the 5^th^ and 95^th^ percentiles.

**Figure 2:**
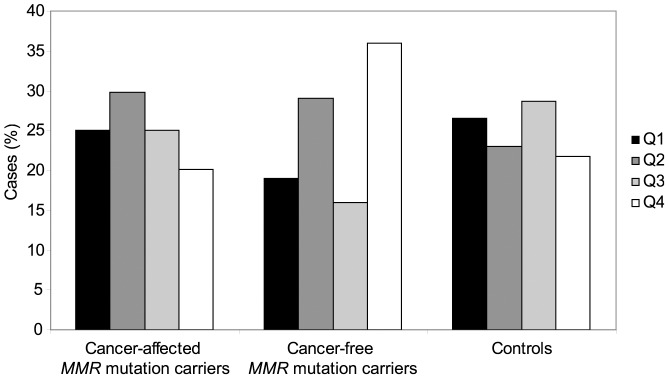
Distribution of cancer-affected and cancer-free *MMR* gene mutation cases and controls by quartiles of age-adjusted telomere length.

Relative telomere length was assessed in 244 *MMR* gene mutation carriers, both cancer-affected (n = 144) and unaffected (n = 100), and in 234 controls, including non-carriers from the same LS families (n = 144) and unrelated cancer-free controls (n = 90) (Figure 1). Relative telomere length values were adjusted for age as indicated in the Materials and Methods section (see statistical analyses). We found that among mutation carriers, cancer cases had significantly shorter age-adjusted telomeres than unaffected individuals (p = 0.032). It is worth noting that unaffected mutation carriers have longer telomeres than unaffected controls: a significant increase of the fourth quartile (longest telomeres) is observed in cancer-free *MMR* mutation carriers (36.0%) compared with controls (21.8%; p = 0.010) and cancer-affected carriers (20.1%; p = 0.009) (Figure 2).

**Figure 3:**
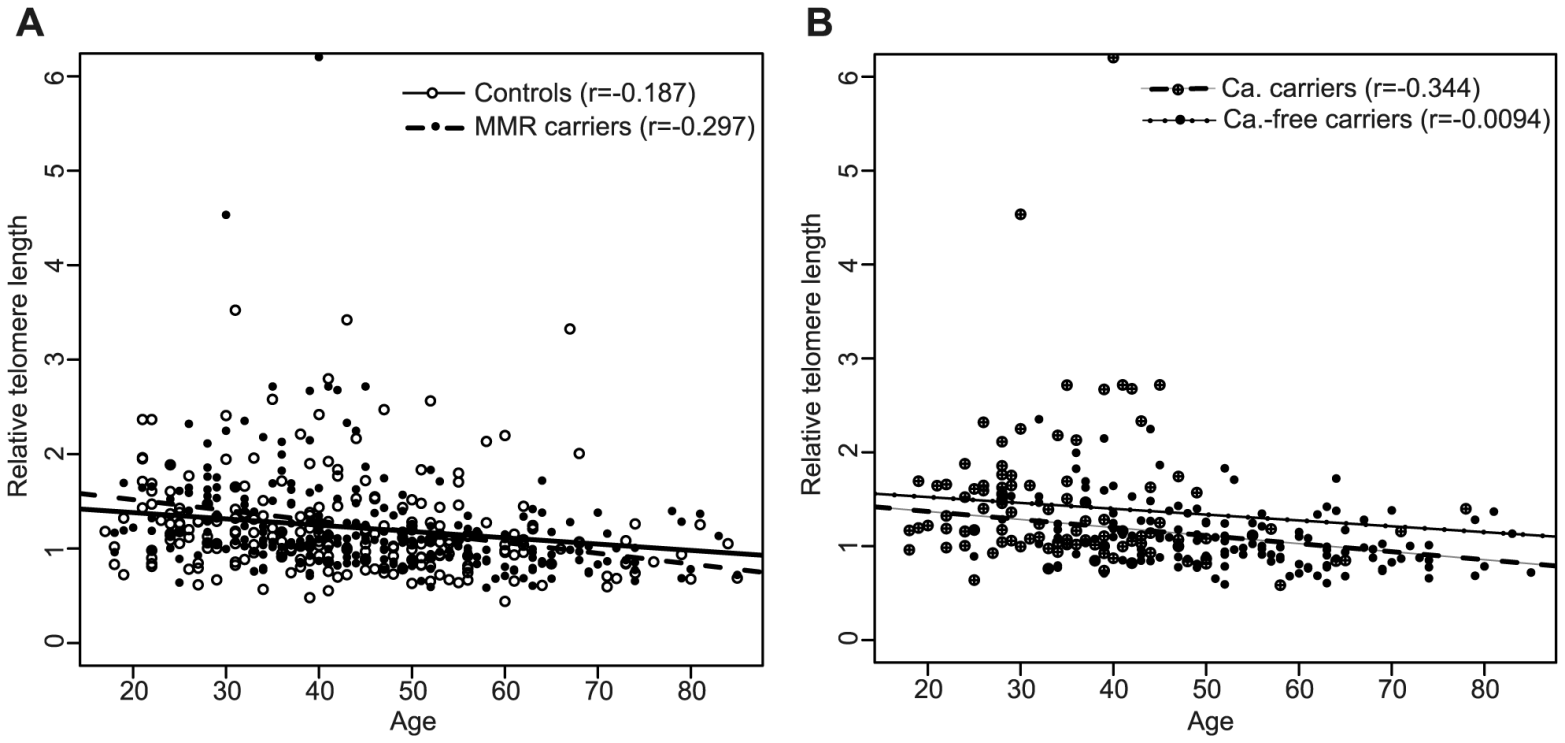
Correlation of relative telomere length (RTL) with age at blood draw. (A) RTL distribution as a function of age for controls (n = 234; white circles) and all *MMR* gene mutation carriers (n = 244; black circles). (B) RTL distribution as a function of age for cancer-affected *MMR* gene mutation carriers (n = 144; black circles) and cancer-free *MMR* gene mutation carriers (n = 100; crossed circles). r: Pearson's correlation coefficient.

As expected, the negative slope of the linear regression line that best fit the RTL data for the controls indicated that telomere length shortened with age (Pearson's correlation coefficient r = −0.187; p = 0.004); the same was observed for the *MMR* gene mutation carriers (r = −0.297; p = 2.37×10^−6^). No differences were observed between both groups (p = 0.204) (Figure 3A). Among *MMR* gene mutation carriers, cancer-affected individuals showed faster telomere attrition with age (r = −0.344; p = 2.4×10^−5^) than cancer-free carriers (r = −0.094; p = 0.351) (for the difference, p = 0.045) (Figure 3B). No differences were observed between *MSH2* (r = −0.446; p = 0.003) and *MLH1* (r = −0.318; p = 0.003) mutation carriers (for the difference, p = 0.435).

### Telomere length and anticipation in the age of cancer onset

**Figure 4:**
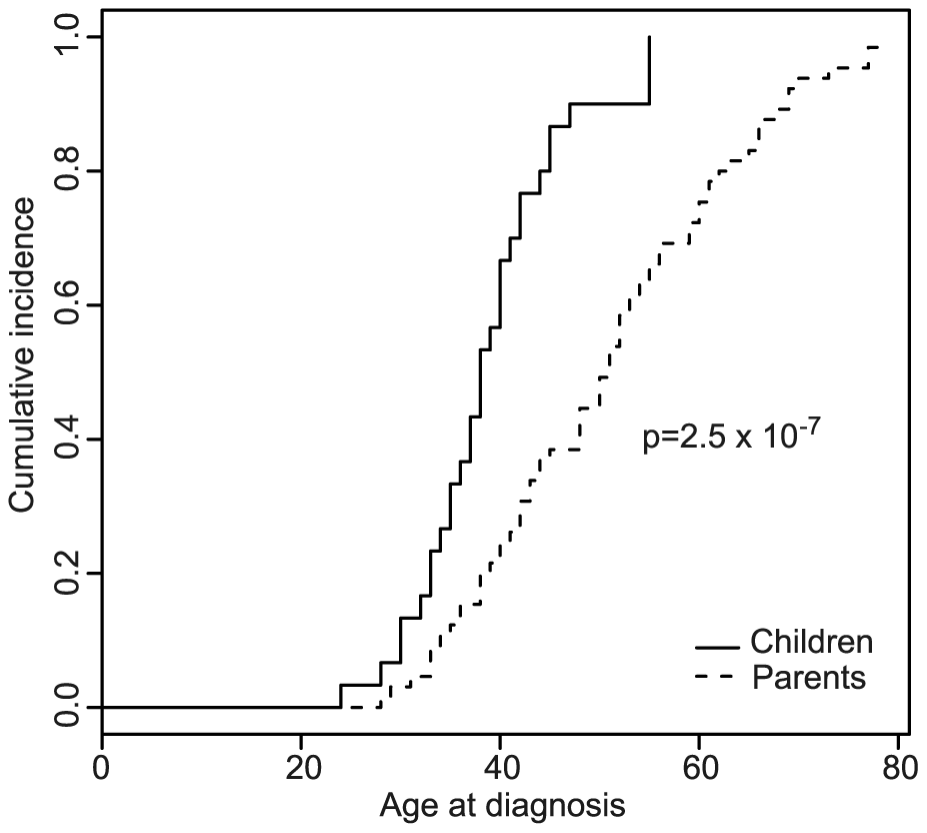
Anticipation in age of cancer onset in LS families. Kaplan-Meier curves and associated p-values showing the differences in age of cancer onset between parents and children, all of them cancer-affected *MMR* gene mutation carriers.

The occurrence of age anticipation in cancer-affected mutation carriers was analyzed in 59 LS families, harbouring *MLH1* (32 families), *MSH2* (20 families) and *MSH6* (7 families) mutations. The distribution of age at cancer diagnosis in parents and children showed a consistent shift to early ages in children (p = 2.5×10^−7^) (Figure 4). On average, cancer was diagnosed 12 years earlier in children (Table 2). Five out of 65 parents and 3 out of 30 children had been diagnosed with cancer (1^st^ LS-related cancer diagnosed) during the follow-up period or clinical surveillance of mutation carriers. Their exclusion from the analysis did not alter the results (data not shown). The information available from polyp removal and surveillance time across generations was incomplete and was not included in the study.

**Table 2:** Differences in average age of onset of cancer (1^st^ LS-related tumor diagnosed) between parents and children with Lynch syndrome.

	Parents				Children						
	N	Age of onset			N	Age of onset			Mean age difference	t-statistic	p-value
		Median	Mean	SD		Median	Mean	SD			
All	65	51.0	50.9	12.8	30	38.0	38.9	7.6	12.0	4.74	7.5×10^−6^
*MLH1*	36	50.5	50.9	11.7	20	38.0	38.0	6.8	12.9	4.53	3.3×10^−5^
*MSH2*	21	53.0	51.4	15.4	7	37.0	41.4	10.4	10	1.59	0.124
*MSH6*	8	44.0	49.6	11.5	2	37.5	37.5	6.4	12.1	1.40	0.200

N: number of subjects; SD: standard deviation.

**Figure 5:**
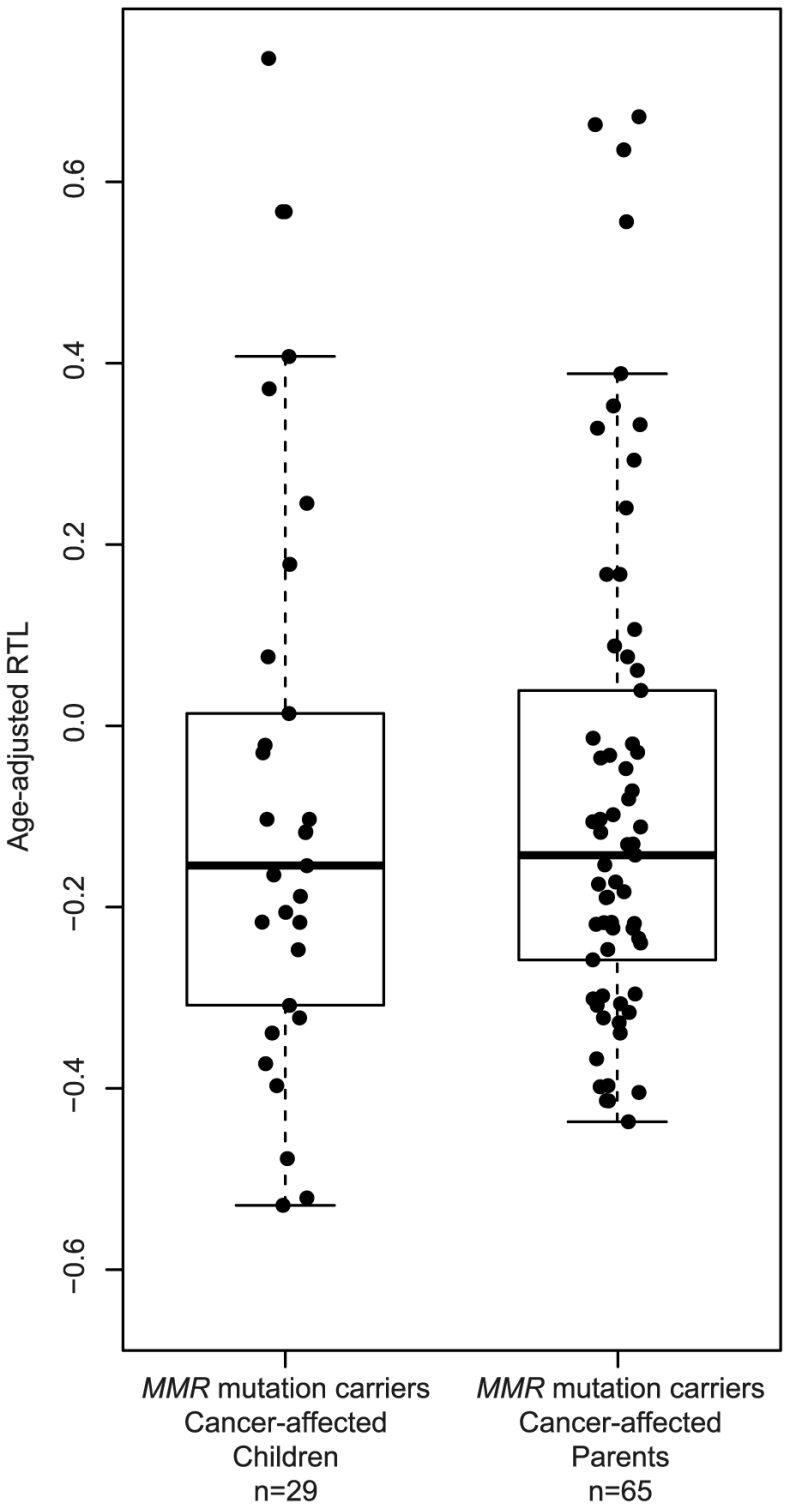
Changes in age-adjusted RTL between parents and children. Box plots representing age-adjusted RTL distributions in cancer-affected parents and children with *MMR* gene mutations. The boxes represent the interquartile range of distributions (25^th^ and 75^th^ percentiles); the horizontal lines within the boxes, the medians; and the vertical lines, the 5^th^ and 95^th^ percentiles.

The relationship between telomere length and anticipation in LS was next investigated. No differences between parents and children were observed in cancer-affected *MMR* gene mutation carriers (p = 0.867) (Figure 5). Similarly, no association was found when *MLH1* and *MSH2* gene mutation cases were analyzed separately (Figure S1). Next, we compared changes in telomere length in individual parent-child pairs (Table 3). In all instances, the child was diagnosed with cancer at an earlier age than the corresponding LS parent. Children showed shorter telomeres than their parents in 10 out of 21 (47.6%) cancer-affected carrier pairs, in 6 out of 9 (66.7%) cancer-free carrier pairs, and in 1 out of 3 (33.3%) cancer-free non-carriers evaluated, although the these differences were not statistically significant (p = 0.562).

**Table 3:** Age-adjusted RTL and anticipation in age at cancer diagnosis in parent-child pairs.

		PARENT				CHILD				
Family	*MMR* gene	Tumor	*Age	Sex	Age-adjusted RTL	Tumor	*Age	Sex	Age-adjusted RTL	**Tel_child_<Tel_parent_
*Cancer-affected carriers*										
Fam-1	*MSH2*	CRC	73	F	−0.172	CRC	45	M	−0.216	yes
Fam-2	*MSH2*	EC	78	F	0.167	CRC	55	F	−0.339	yes
Fam-2	*MSH2*	CRC	55	F	−0.339	CRC	33	F	−0.188	no
Fam-2	*MSH2*	EC	78	F	0.167	CRC	55	F	−0.217	yes
Fam-3	*MSH2*	CRC	60	F	−0.098	CRC	35	F	0.407	no
Fam-4	*MLH1*	CRC	53	F	−0.143	CRC	40 (in surv)	F	0.372	no
Fam-4	*MLH1*	CRC	53	F	−0.143	CRC	32	M	−0.521	yes
Fam-5	*MLH1*	CRC	48	M	0.328	CRC	35	M	0.567	no
Fam-6	*MLH1*	CRC	52	F	0.635	CRC	34	F	0.736	no
Fam-7	*MLH1*	CRC	50	F	0.388	CRC	55 (in surv)	F	−0.103	yes
Fam-8	*MLH1*	CRC	51	M	−0.240	CRC	28	F	−0.022	no
Fam-9	*MLH1*	CRC	51	M	0.332	CRC	45	F	0.178	yes
Fam-10	*MLH1*	CRC	33	M	−0.398	CRC	24	F	−0.154	no
Fam-11	*MLH1*	CRC	69 (in surv)	M	−0.298	CRC	37	F	−0.206	no
Fam-11	*MLH1*	CRC	69 (in surv)	M	−0.298	CRC	41 (in surv)	F	−0.247	no
Fam-12	*MLH1*	CRC	66	F	−0.014	CRC	47	M	−0.373	yes
Fam-12	*MLH1*	CRC	66	F	−0.014	CRC	39	M	−0.322	yes
Fam-12	*MLH1*	CRC	66	F	−0.014	CRC	40	M	−0.103	yes
Fam-13	*MLH1*	CRC	60	F	−0.081	CRC	38	F	−0.030	no
Fam-14	*MLH1*	CRC	77	F	−0.235	CRC	38	F	−0.076	no
Fam-15	*MSH6*	UC	69	M	−0.032	CRC	33	M	−0.478	yes
*Cancer-free carriers*										
Fam-16	*MLH1*	-	48	M	−0.347	-	24	M	−0.327	no
Fam-16	*MLH1*	-	48	M	−0.347	-	25	F	−0.688	yes
Fam-17	*MLH1*	-	47	M	−0.121	-	27	F	−0.387	yes
Fam-18	*MLH1*	-	64	F	−0.244	-	36	M	−0.095	no
Fam-18	*MLH1*	-	64	F	−0.244	-	39	M	−0.134	no
Fam-19	*MLH1*	-	45	F	0.048	-	18	M	−0.202	yes
Fam-20	*MLH1*	-	78	M	0.399	-	37	F	−0.186	yes
Fam-21	*MSH2*	-	47	F	0.552	-	22	M	−0.021	yes
Fam-22	*MSH6*	-	65	F	−0.230	-	30	M	−0.300	yes
*** *Cancer-free non-carriers*										
Fam-23	-	-	71	F	−0.336	-	45	M	0.000	no
Fam-20	-	-	74	F	−0.284	-	50	M	−0.181	no
Fam-24	-	-	85	F	−0.268	-	53	F	−0.432	yes

MMR: DNA mismatch repair; RTL: relative telomere length; CRC: colorectal cancer; EC: endometrial cancer; UC: urinary tract cancer; M: male; F: female; in surv: cancer diagnosed as a consequence of extensive clinical surveillance of LS families.

* Age: Age at cancer diagnosis for cancer-afected carriers, and age at blood draw for cancer-free carriers and non-carriers

** Tel_child_<Tel_parent_, age-adjusted telomere length is shorter in the child than in the parent. “Yes” indicates that age-adjusted RTL is smaller in the child than in the parent, therefore indicating association with anticipation when cancer-affected carriers are studied.

*** Cancer-free non-carriers are individuals who belong to Lynch syndrome families but do not carry the pathogenic *MMR* gene mutation.

Anticipation was associated with shorter telomeres in all (5/5) mother-son cancer-affected *MMR* gene mutation carriers, whereas this association was only observed in 31% (5/16) of the other cancer-affected carrier pair combinations (p = 0.012) (Table 3).

## Discussion

In this study we found that cancer-affected *MMR* gene mutation carriers have shorter telomeres and faster telomere attrition rates (accelerated telomere shortening with age) than unaffected mutation carriers and controls, and that telomere shortening is not associated with anticipation in the age of cancer onset in successive generations.

In line of recent retrospective and prospective studies on sporadic CRC risk and leukocyte telomere length, it has been suggested that the shorter telomere length observed in retrospectively collected samples from CRC patients is not a cancer risk factor, but rather a consequence of the disease (from treatment or disease burden) [16], [17]. However, a recent prospective study has shown that both extremely long and extremely short telomeres increase CRC risk [18]. In hereditary cases, we observe shorter telomeres in cancer-affected *MMR* gene mutation carriers than in cancer-free carriers, suggesting either that the shortened telomeres are a result of the disease, or that shorter telomere length is an additional risk factor for LS patients, i.e. a risk modifier. This is supported by our recent observation that a variant in the telomerase gene, *hTERT*, increases cancer risk in young LS patients (<45 years of age), as it is associated with shortened telomeres in cancer-affected *MMR* gene mutation carriers [11]. Further studies assessing telomere length before and after cancer diagnosis in CRC patients will provide a definitive answer regarding the suggested effect of cancer on telomere length in blood cells. Unfortunately, samples from sporadic CRC cases that underwent the same DNA extraction and storage than the hereditary cases and controls herein studied were not available, precluding a direct comparison. This study will be critical to clarify whether telomere shortening is specific to cancer-affected LS patients or if it occurs in all CRC-affected patients.

Our data show longer telomeres in cancer-free *MMR* gene mutation carriers compared to cancer-free controls. A plausible explanation for this observation, and consistent with the fact that short telomeres act as factors of increased cancer risk, is that longer telomeres protect *MMR* gene mutation carriers from developing cancer. On the other hand, Jones et al. proposed that certain genetic factors that increase CRC risk may cause longer telomeres [17]. Alternatively, and in line with this model, our results might suggest that MMR haploinsufficiency, as a genetic factor of CRC risk, can exert a lengthening effect on the telomeres. Additional studies to assess the effect of MMR haploinsufficiency on telomere lengthening are needed to validate this hypothesis.

We also observed that leukocyte telomere dynamics differ between cancer-free mutation carriers and cancer-affected carriers, the latter group showing faster telomere shortening with age. Bozzao et al. found that affected carriers showed accelerated attrition rate compared to controls [28]. Based on previously published data on MMR-deficiency and telomeres [29]–[33], they suggested that MMR haploinsuffiency in LS individuals may lead to tolerance of short telomeres, causing faster telomere shortening over the course of life. However, our results show that this increased shortening rate does not occur in cancer-free carriers, thus invalidating the hypothesis. Therefore, this effect may be either the result of telomere shortening in cancer-affected individuals, or a marker of increased cancer risk among *MMR* gene mutation carriers, supporting the idea that short telomere length or accelerated telomere attrition rate are factors of increased cancer risk in LS. Identifying the dynamics of leukocyte telomere length with age in sporadic cases from retrospective and prospective studies may resolve this question.

No association was found between the anticipation in the age of onset observed in successive generations and telomere length. For years, and despite the numerous reports identifying anticipation in LS, it was uncertain whether true genetic anticipation contributed to the early diagnosis age observed in LS. Recently, a Bayesian method that corrects for random effects, isolating the confounding effect of changes in secular trends, screening, and medical practices, and adjusting for changes in age-specific incidence across birth cohorts, confirmed the anticipation in the age of onset between successive generations of LS families [10]. Nevertheless, the molecular mechanism underlying this observation has not been yet identified. Due to the lack of information on surveillance time in parents and children, polyp removal, and other confounding factors, as well as the insufficient sample size for an accurate assessment of genetic anticipation, it is certain that the measurement obtained in our series (12 years) is clearly overestimated. Nevertheless, we accept the results of previous reports on this matter in which accurate statistical methodologies were used and anticipation was still confirmed [8]–[10], and our series was used to assess the involvement of telomere attrition in anticipation. Our data rule out the role of telomere shortening in anticipation in LS. As previously suggested, other molecular mechanisms such as the accumulation of mismatch repair slippage events through generations or other genetic or environmental factors might explain age anticipation in successive generations of LS families [34], [35]. Considering the limitations of our study regarding sample size, and the inconclusive results obtained when studying telomere length in cancer patients, further studies on larger LS series should be performed to validate these results.

Van Vliet et al. recently suggested that CRC risk in carriers of *MMR* gene mutations depends on the parent-of-origin of the mutation. In particular, they suggested a maternally transmitted mechanism modifying cancer risk in male *MMR* gene mutation carriers [36]. Here we observe anticipation associated with shorter telomeres in all mother-son cancer-affected *MMR* gene mutation carriers, whereas the same association is only found in 31% of the other cancer-affected carrier pair combinations. Although the sample size of parent-child pairs is very limited, this observation might suggest that the maternally transmitted risk mechanism is shortened telomeres. Studies assessing whether telomere shortening occurs in the female germline, as recently demonstrated in other mammals [37], together with the analysis of larger samples of LS parent-child pairs, are required to verify this hypothesis.

In conclusion, our findings indicate that cancer-affected *MMR* gene mutation carriers show distinct features and dynamics of telomere length measured in blood DNA, to controls and unaffected mutation carriers. While we rule out telomere length attrition as the common cause of anticipation in LS, it may account for the dependence of cancer risk on the parent-of-origin of *MMR* gene mutations previously observed.

## Supporting Information

Figure S1 **Age-adjusted RTL distributions in cancer-affected parents and children with** ***MLH1*** **and** ***MSH2*** **gene mutations.** The boxes represent the interquartile range of distributions (25^th^ and 75^th^ percentiles); the horizontal lines within the boxes, the medians; and the vertical lines, the 5^th^ and 95^th^ percentiles. Pairwise comparisons using Wilcoxon rank sum test showed no differences in RTL distributions between cancer-affected parents and children with germline mutations in *MLH1* (p = 0.65), and between parents and children with mutations in *MSH2* (p = 0.67).
(TIF)

## References

[1] Lynch HT, Smyrk TC, Watson P, Lanspa SJ, Lynch JF, et al (1993) Genetics, natural history, tumor spectrum, and pathology of hereditary nonpolyposis colorectal cancer: an updated review. Gastroenterology 104: 1535–1549. 8482467 10.1016/0016-5085(93)90368-m

[2] Scott RJ, McPhillips M, Meldrum CJ, Fitzgerald PE, Adams K, et al (2001) Hereditary nonpolyposis colorectal cancer in 95 families: differences and similarities between mutation-positive and mutation-negative kindreds. Am J Hum Genet 68: 118–127. 11112663 10.1086/316942 PMC1234904

[3] Vasen HF, Taal BG, Griffioen G, Nagengast FM, Cats A, et al (1994) Clinical heterogeneity of familial colorectal cancer and its influence on screening protocols. Gut 35: 1262–1266. 7959234 10.1136/gut.35.9.1262 PMC1375704

[4] Rodriguez-Bigas MA, Lee PH, O'Malley L, Weber TK, Suh O, et al (1996) Establishment of a hereditary nonpolyposis colorectal cancer registry. Dis Colon Rectum 39: 649–653. 8646951 10.1007/BF02056944

[5] Westphalen AA, Russell AM, Buser M, Berthod CR, Hutter P, et al (2005) Evidence for genetic anticipation in hereditary non-polyposis colorectal cancer. Hum Genet 116: 461–465. 15772852 10.1007/s00439-005-1272-5

[6] Stella A, Surdo NC, Lastella P, Barana D, Oliani C, et al (2007) Germline novel MSH2 deletions and a founder MSH2 deletion associated with anticipation effects in HNPCC. Clin Genet 71: 130–139. 17250661 10.1111/j.1399-0004.2007.00745.x

[7] Nilbert M, Timshel S, Bernstein I, Larsen K (2009) Role for genetic anticipation in Lynch syndrome. J Clin Oncol 27: 360–364. 19075283 10.1200/JCO.2008.16.1281

[8] Larsen K, Petersen J, Bernstein I, Nilbert M (2009) A parametric model for analyzing anticipation in genetically predisposed families. Stat Appl Genet Mol Biol 8: Article26. 19492984 10.2202/1544-6115.1424

[9] Boonstra PS, Gruber SB, Raymond VM, Huang SC, Timshel S, et al (2010) A review of statistical methods for testing genetic anticipation: looking for an answer in Lynch syndrome. Genet Epidemiol 34: 756–768. 20878717 10.1002/gepi.20534 PMC3894615

[10] Boonstra PS, Mukherjee B, Taylor JM, Nilbert M, Moreno V, et al (2011) Bayesian modeling for genetic anticipation in presence of mutational heterogeneity: a case study in Lynch syndrome. Biometrics 67: 1627–1637. 21627626 10.1111/j.1541-0420.2011.01607.x PMC3176998

[11] Bellido F, Guino E, Jagmohan-Changur S, Segui N, Pineda M, et al (2012) Genetic variant in the telomerase gene modifies cancer risk in Lynch syndrome. Eur J Hum Genet 10.1038/ejhg.2012.204 PMC3641380 22948024

[12] Londono-Vallejo JA (2004) Telomere length heterogeneity and chromosome instability. Cancer Lett 212: 135–144. 15341022 10.1016/j.canlet.2004.05.008

[13] Meeker AK, Hicks JL, Iacobuzio-Donahue CA, Montgomery EA, Westra WH, et al (2004) Telomere length abnormalities occur early in the initiation of epithelial carcinogenesis. Clin Cancer Res 10: 3317–3326. 15161685 10.1158/1078-0432.CCR-0984-03

[14] Ma H, Zhou Z, Wei S, Liu Z, Pooley KA, et al (2011) Shortened telomere length is associated with increased risk of cancer: a meta-analysis. PLoS One 6: e20466. 21695195 10.1371/journal.pone.0020466 PMC3112149

[15] Wentzensen IM, Mirabello L, Pfeiffer RM, Savage SA (2011) The association of telomere length and cancer: a meta-analysis. Cancer Epidemiol Biomarkers Prev 20: 1238–1250. 21467229 10.1158/1055-9965.EPI-11-0005 PMC3111877

[16] Prescott J, Wentzensen IM, Savage SA, De Vivo I (2012) Epidemiologic evidence for a role of telomere dysfunction in cancer etiology. Mutat Res 730: 75–84. 21756922 10.1016/j.mrfmmm.2011.06.009 PMC3222719

[17] Jones AM, Beggs AD, Carvajal-Carmona L, Farrington S, Tenesa A, et al (2012) TERC polymorphisms are associated both with susceptibility to colorectal cancer and with longer telomeres. Gut 61: 248–254. 21708826 10.1136/gut.2011.239772 PMC3245900

[18] Cui Y, Cai Q, Qu S, Chow WH, Wen W, et al (2012) Association of Leukocyte Telomere Length with Colorectal Cancer Risk: Nested Case-Control Findings from the Shanghai Women's Health Study. Cancer Epidemiol Biomarkers Prev 21: 1807–1813. 22911335 10.1158/1055-9965.EPI-12-0657 PMC3467322

[19] Vulliamy T, Marrone A, Szydlo R, Walne A, Mason PJ, et al (2004) Disease anticipation is associated with progressive telomere shortening in families with dyskeratosis congenita due to mutations in TERC. Nat Genet 36: 447–449. 15098033 10.1038/ng1346

[20] Marrone A, Walne A, Dokal I (2005) Dyskeratosis congenita: telomerase, telomeres and anticipation. Curr Opin Genet Dev 15: 249–257. 15917199 10.1016/j.gde.2005.04.004

[21] Armanios M, Chen JL, Chang YP, Brodsky RA, Hawkins A, et al (2005) Haploinsufficiency of telomerase reverse transcriptase leads to anticipation in autosomal dominant dyskeratosis congenita. Proc Natl Acad Sci U S A 102: 15960–15964. 16247010 10.1073/pnas.0508124102 PMC1276104

[22] Tabori U, Nanda S, Druker H, Lees J, Malkin D (2007) Younger age of cancer initiation is associated with shorter telomere length in Li-Fraumeni syndrome. Cancer Res 67: 1415–1418. 17308077 10.1158/0008-5472.CAN-06-3682

[23] Trkova M, Prochazkova K, Krutilkova V, Sumerauer D, Sedlacek Z (2007) Telomere length in peripheral blood cells of germline TP53 mutation carriers is shorter than that of normal individuals of corresponding age. Cancer 110: 694–702. 17567834 10.1002/cncr.22834

[24] Gramatges MM, Telli ML, Balise R, Ford JM (2010) Longer relative telomere length in blood from women with sporadic and familial breast cancer compared with healthy controls. Cancer Epidemiol Biomarkers Prev 19: 605–613. 20142254 10.1158/1055-9965.EPI-09-0896

[25] Martinez-Delgado B, Yanowsky K, Inglada-Perez L, Domingo S, Urioste M, et al (2011) Genetic anticipation is associated with telomere shortening in hereditary breast cancer. PLoS Genet 7: e1002182. 21829373 10.1371/journal.pgen.1002182 PMC3145621

[26] Cawthon RM (2009) Telomere length measurement by a novel monochrome multiplex quantitative PCR method. Nucleic Acids Res 37: e21. 19129229 10.1093/nar/gkn1027 PMC2647324

[27] Iwama H, Ohyashiki K, Ohyashiki JH, Hayashi S, Yahata N, et al (1998) Telomeric length and telomerase activity vary with age in peripheral blood cells obtained from normal individuals. Hum Genet 102: 397–402. 9600234 10.1007/s004390050711

[28] Bozzao C, Lastella P, Ponz de Leon M, Pedroni M, Di Gregorio C, et al (2011) Analysis of telomere dynamics in peripheral blood cells from patients with Lynch syndrome. Cancer 117: 4325–4335. 21387278 10.1002/cncr.26022

[29] Pickett HA, Baird DM, Hoff-Olsen P, Meling GI, Rognum TO, et al (2004) Telomere instability detected in sporadic colon cancers, some showing mutations in a mismatch repair gene. Oncogene 23: 3434–3443. 15048084 10.1038/sj.onc.1207477

[30] Mendez-Bermudez A, Hills M, Pickett HA, Phan AT, Mergny JL, et al (2009) Human telomeres that contain (CTAGGG)n repeats show replication dependent instability in somatic cells and the male germline. Nucleic Acids Res 37: 6225–6238. 19656953 10.1093/nar/gkp629 PMC2764434

[31] Martinez P, Siegl-Cachedenier I, Flores JM, Blasco MA (2009) MSH2 deficiency abolishes the anticancer and pro-aging activity of short telomeres. Aging Cell 8: 2–17. 18986375 10.1111/j.1474-9726.2008.00441.x

[32] Rampazzo E, Bertorelle R, Serra L, Terrin L, Candiotto C, et al (2010) Relationship between telomere shortening, genetic instability, and site of tumour origin in colorectal cancers. Br J Cancer 102: 1300–1305. 20386541 10.1038/sj.bjc.6605644 PMC2856015

[33] Mendez-Bermudez A, Royle NJ (2011) Deficiency in DNA mismatch repair increases the rate of telomere shortening in normal human cells. Hum Mutat 32: 939–946. 21538690 10.1002/humu.21522

[34] Gruber SB, Mukherjee B (2009) Anticipation in lynch syndrome: still waiting for the answer. J Clin Oncol 27: 326–327. 19075261 10.1200/JCO.2008.19.1445

[35] Coolbaugh-Murphy MI, Xu JP, Ramagli LS, Ramagli BC, Brown BW, et al (2010) Microsatellite instability in the peripheral blood leukocytes of HNPCC patients. Hum Mutat 31: 317–324. 20052760 10.1002/humu.21190 PMC3544178

[36] Van Vliet CM, Dowty JG, van Vliet JL, Smith L, Mead LJ, et al (2011) Dependence of colorectal cancer risk on the parent-of-origin of mutations in DNA mismatch repair genes. Hum Mutat 32: 207–212. 21120946 10.1002/humu.21408 PMC3228833

[37] Bender HS, Murchison EP, Pickett HA, Deakin JE, Strong MA, et al (2012) Extreme telomere length dimorphism in the tasmanian devil and related marsupials suggests parental control of telomere length. PLoS One 7: e46195. 23049977 10.1371/journal.pone.0046195 PMC3458001

