# Supplementary figures and images for: Telomere Length and Genetic Anticipation in Lynch Syndrome
Source: PLoS One. 2013 Apr 23;8(4):e61286. doi: 10.1371/journal.pone.0061286 (PMC3634050; doi:10.1371/journal.pone.0061286)

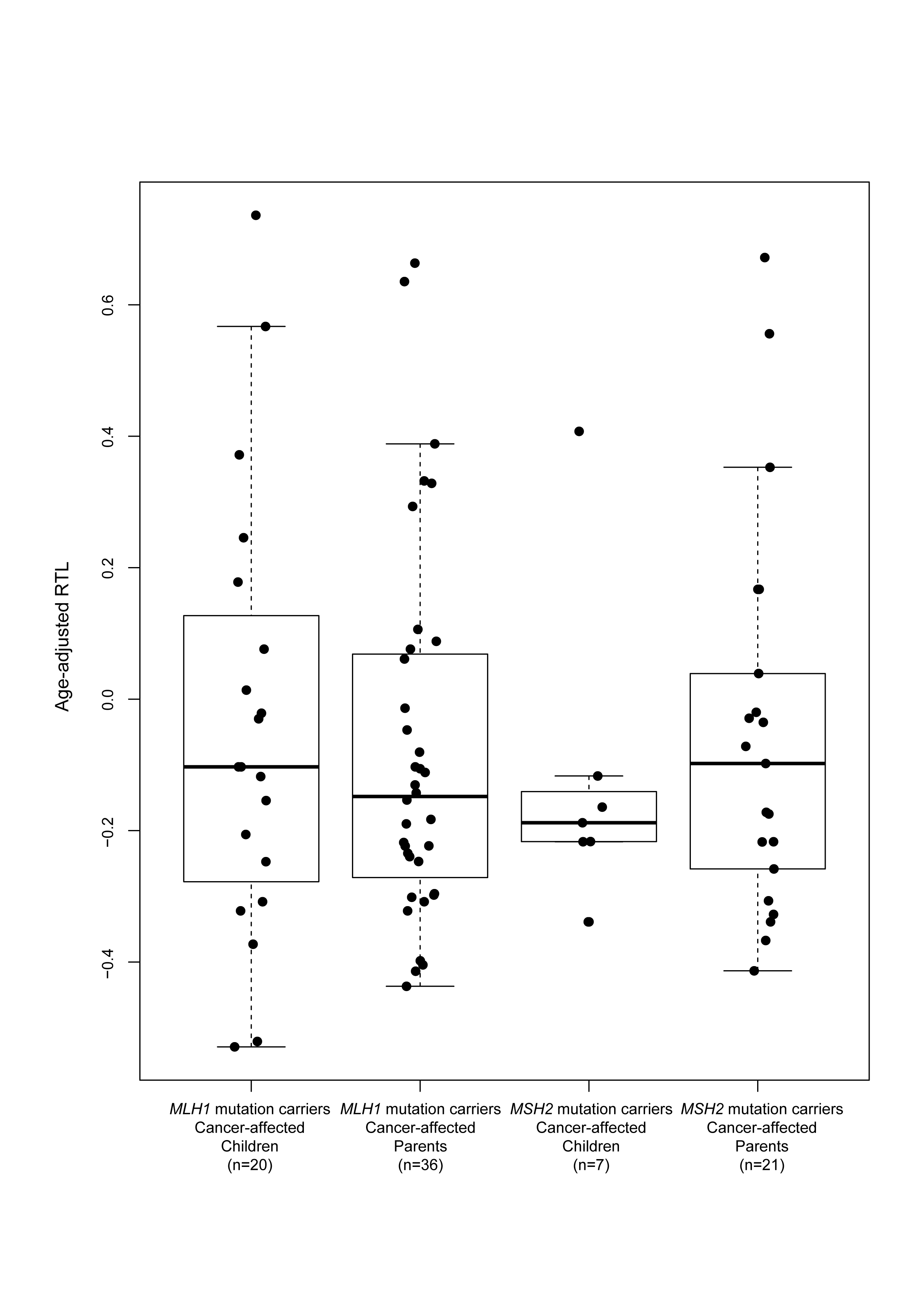

Supplement: Figure S1 — Age-adjusted RTL distributions in cancer-affected parents and children with MLH1 and MSH2 gene mutations. The boxes represent the interquartile range of distributions (25th and 75th percentiles); the horizontal lines within the boxes, the medians; and the vertical lines, the 5th and 95th percentiles. Pairwise comparisons using Wilcoxon rank sum test showed no differences in RTL distributions between cancer-affected parents and children with germline mutations in MLH1 (p = 0.65), and between parents and children with mutations in MSH2 (p = 0.67). (TIF) [file pone.0061286.s001.tif]
